# Multivisceral resection for adenocarcinoma of the pancreatic body and tail—a retrospective single-center analysis

**DOI:** 10.1186/s12957-020-01973-x

**Published:** 2020-08-20

**Authors:** Oliver Beetz, Akin Sarisin, Alexander Kaltenborn, Jürgen Klempnauer, Michael Winkler, Gerrit Grannas

**Affiliations:** Department of General, Visceral and Transplant Surgery, Hannover Medical School, Carl-Neuberg-Strasse 1, 30625 Hannover, Germany

**Keywords:** Left-sided pancreatic cancer, Distal pancreatectomy, Multivisceral resection, Extended surgery, Lymph node ratio, Pancreatic fistula

## Abstract

**Background:**

Adenocarcinoma of the pancreatic body and tail is associated with a dismal prognosis. As patients frequently present themselves with locally advanced tumors, extended surgery including multivisceral resection is often necessary in order to achieve tumor-free resection margins. The aim of this study was to identify prognostic factors for postoperative morbidity and mortality and to evaluate the influence of multivisceral resections on patient outcome.

**Methods:**

This is a retrospective analysis of 94 patients undergoing resection of adenocarcinoma located in the pancreatic body and/or tail between April 1995 and December 2016 at our institution. Uni- and multivariable Cox regression analysis was conducted to identify independent prognostic factors for postoperative survival.

**Results:**

Multivisceral resections, including partial resections of the liver, the large and small intestines, the stomach, the left kidney and adrenal gland, and major vessels, were carried out in 47 patients (50.0%). The median postoperative follow-up time was 12.90 (0.16–220.92) months.

Median Kaplan-Meier survival after resection was 12.78 months with 1-, 3-, and 5-year survival rates of 53.2%, 15.8%, and 9.0%. Multivariable Cox regression identified coeliac trunk resection (*p* = 0.027), portal vein resection (*p* = 0.010), intraoperative blood transfusions (*p* = 0.005), and lymph node ratio in percentage (*p* = 0.001) as independent risk factors for survival. Although postoperative complications requiring surgical revision were observed more frequently after multivisceral resections (14.9 versus 2.1%; *p* = 0.029), postoperative survival was not significantly inferior when compared to patients undergoing standard distal or subtotal pancreatectomy (12.35 versus 13.87 months; *p* = 0.377).

**Conclusions:**

Our data indicates that multivisceral resection in cases of locally advanced pancreatic carcinoma of the body and/or tail is justified, as it is not associated with increased mortality and can even facilitate long-term survival, albeit with an increase in postoperative morbidity. Simultaneous resections of major vessels, however, should be considered carefully, as they are associated with inferior survival.

## Background

Pancreatic adenocarcinoma is a fatal malignant disease with 5-year survival rates below 10% and an increasing incidence worldwide [1]. As typical symptoms often occur at a late stage, 80 to 90% of the patients present themselves with unresectable tumors and dismal prognosis despite efforts of improving non-surgical therapies including chemotherapy regimens [2, 3].

In case of resection, severable risk factors impeding long-term survival have been identified in the past, including lymph node metastases, advanced tumor stage, positive resection margins, and distant disease, among others [4, 5].

Although the influence of the tumor localization has been a matter of great debate, data from large patient registries have demonstrated that lesions of the pancreatic body and tail, accounting for around 20 to 25% of the pancreatic adenocarcinomas, are associated with inferior survival, most likely as a result of a delayed diagnosis and a more aggressive tumor biology [6–9]. Distal pancreatectomy is the standard procedure for these tumors; however, advanced tumor stages often require additional resection of extrapancreatic tissue, including large vessels. Data on the effect of such resections is scarce. Recently, Malinka et al. and Panzeri et al. each published results of single-center studies, indicating that multivisceral resections are justifiable in selected patients [10, 11]. However, especially the role of vascular resections remains to be elusive. Recent recommendations, for example, discourage arterial resections of the coeliac axis without prior neoadjuvant chemotherapy, whereas venous resections are generally regarded as feasible and safe. Nonetheless, reports are contradictory throughout the available literature, not least because data of patients with pancreatic head and body/tail lesions are often pooled [12, 13].

The aim of our study was to evaluate the influence of different types of multivisceral resections, among other potential prognostic factors, on postoperative morbidity and mortality in patients with adenocarcinoma of the pancreatic body and/or tail.

## Methods

### Study design and patient cohort

This is a retrospective, single-center analysis of 94 patients with adenocarcinoma of the pancreatic body and/or tail undergoing surgery between April 1995 and December 2016 at the Department of General, Visceral and Transplant Surgery, Hannover Medical School, in Hannover, Germany.

### Inclusion and exclusion criteria

Included were all patients with intraoperatively and histologically confirmed ductal adenocarcinoma of the pancreatic body and/or tail undergoing resection. No exclusion criteria were defined.

### Definition of variables

Multivisceral resections were defined as distal or subtotal pancreatectomy (including lymphadenectomy of the stations 10, 11, and 18 and splenectomy) with additional resection of contiguous or distant organs, including large vessels, also referred to as “extended distal pancreatectomy” and “distal pancreatectomy with resection of non-contiguous organs” according to the International Study Group for Pancreatic Surgery [14, 15].

For classification of medical co-morbidities and preoperative fitness prior surgery, the American Society of Anesthesiologists (ASA) Physical Status Classification System was applied [16].

Preoperative anemia was defined according to the World Health Organization as hemoglobin concentrations lower than 12.0 g/dl in female patients and lower than 13.0 g/dl in male patients [17].

Surgical complications were defined as postoperative complications requiring surgical revision or intervention.

Postoperative pancreatic fistulas were defined according to the latest definition of the International Study Group for Pancreatic Surgery [18]. Postoperative pancreatic fistulas grade B and C were defined as clinically relevant.

For the classification of the respective tumor stage, the current AJCC/UICC 8^th^ edition was applied [19].

The lymph node ratio was analyzed as continuous variable in percent for regression analysis. For visualization of postoperative survival, the lymph node ratio was analyzed as binary variable (cut-off ≥ 20%).

Follow-up time was defined as time between surgery and last contact or death.

Survival times are reported as Kaplan-Meier median estimates, unless stated otherwise.

### Study endpoints

The primary endpoint was postoperative survival after pancreatic resection. Secondary endpoints were clinically relevant postoperative pancreatic fistulas and postoperative complications in general.

### Statistical analysis

The influence of nominal and ordinal variables on binary endpoints was analyzed with chi-squared test and Fisher’s exact test where appropriate.

Continuous endpoints, such as mean and median values, were compared with the Student’s *t* test in case of normal distribution or the Mann-Whitney *U* test.

Risk factors for postoperative survival were analyzed with Cox regression analysis. Independent risk factors were identified by purposeful selection of variables with a rate of missing values of < 10% and *p* values in univariable Cox regression of < 0.300 and consecutive stepwise forward selection. Kaplan-Meier analyses including Log-rank tests were performed where appropriate.

The identification of risk factors for clinically relevant postoperative pancreatic fistulas was achieved by univariable and multivariable binary logistic regression analysis, as described above.

Statistical significance was set at a *p* value of < 0.050 and is shown in bold (tables) or marked with an asterisk (figures).

The collected data was implemented and analyzed using SPSS statistical software (version 26; SPSS Inc.; IBM Corporation, Armonk, NY USA), and respective figures were created with GraphPad Prism (version 8.4.0 for Windows, GraphPad Software, La Jolla, CA USA).

## Results

### Study cohort and preoperative course

Females (51.1%) and males (48.9%) were equally distributed among the cohort of 94 patients. The median age was 65 (41–84) years.

Patients most commonly presented themselves with epigastric pain (68.1%), weight loss (39.4%), and back pain (20.2%). Diabetes prior surgery was present in 20 (21.3%) patients.

Elevated tumor markers (CA19-9 and CEA) were observed in 42 (44.7%) and 19 (20.2%) patients, respectively, with a high rate of missing values, especially in the early observation period.

Twenty-two (23.4%) patients showed severe systemic disease and/or substantive functional limitations prior surgery (i.e., ASA score > 2).

A summary of the biometrical and preoperative data is provided in Table 1.

**Table 1:** Descriptive statistics of the study cohort undergoing distal pancreatectomy for adenocarcinoma

Variables			n_**abs**_ (n_**%**_)	Mean; Median (Range)	Missing values n (%)
**Biometrics**	Female gender		48 (51.1)		0 (0.0)
	Age (in years)			65.0; 65.0 (41-84)	0 (0.0)
**Preoperative Course**	Initial symptoms	Epigastric pain	64 (68.1)		4 (4.3)
		Weight loss	37 (39.4)		
		Back pain	19 (20.2)		
		Inappetence	13 (13.8)		
		Nausea	12 (12.8)		
		Vomiting	6 (6.4)		
		Fatigue	6 (6.4)		
		Others	29 (30.9)		
	ASA score	1	7 (7.4)		16 (17.0)
		2	49 (52.1)		
		3	22 (23.4)		
	Diabetes		20 (21.3)		3 (3.2)
	Diagnostics	Hemoglobin (in g/dl)		13.4; 13.3 (9.9-17.2)	2 (2.1)
		Anemia	14 (14.9)		
		Platelets (in 10³ per μl)		237.85; 236 (56-551)	2 (2.1)
		Elevated CA19-9	42 (44.7)		31 (33.0)
		Elevated CEA	19 (20.2)		34 (36.2)
		CT scan	82 (87.2)		4 (4.3)
		MRI scan	5 (5.3)		4 (4.3)
		Ultrasound	74 (78.7)		13 (13.8)
	Preoperative hospital stay (in days)			2.82; 2 (1-20)	1 (1.1)
**Surgical details**	Type of pancreatectomy	Distal	85 (90.4)		0 (0.0)
		Subtotal	9 (9.6)		
	Splenectomy		93 (98.9)		0 (0.0)
	Multivisceral resection		47 (50.0)		0 (0.0)
	Including (partial) resection of	Liver	12 (12.8)		
		Large intestine	12 (12.8)		
		Small intestine	8 (8.5)		
		Stomach	14 (14.9)		
		Kidney	5 (5.3)		
		Adrenal gland	13 (13.8)		
		Coeliac trunk	5 (5.3)		
		Portal vein	15 (16.0)		
	Intraoperative PRBC		34 (36.2)		0 (0.0)
	Intraoperative PRBC (n)			1.34; 0 (0-24)	
	Intraoperative FFP		15 (16.0)		0 (0.0)
	Operation time (in minutes)			165.62; 155 (68-360)	2 (2.1)
**Histopathological results**	Tumor localization	Tail	42 (44.7)		0 (0.0)
		Body	40 (42.6)		
		Tail and body	12 (12.8)		
		Including body	52 (55.3)		
	Invasion of peripancreatic tissue		78 (83.0)		0 (0.0)
	T staging	Tumor size (in cm)		4.79; 4.5 (1.0-14.0)	0 (0.0)
		1	7 (7.4)		0 (0.0)
		2	37 (39.4)		
		3	45 (47.9)		
		4	5 (5.3)		
		T stage ≥ 3	50 (53.2)		
	Lymph node status	Lymph nodes (*n* total)		11.86; 10 (1-36)	3 (3.2)
		Lymph nodes (*n* positive)		1.22; 0 (0-8)	
		Lymph node ratio (in %)		11.68; 0 (0-100)	
		Lymph node ratio ≥ 20%	24 (25.5)		
		N 0 stage	49 (52.1)		2 (2.1)
		N 1 stage	34 (36.2)		
		N 2 stage	9 (9.6)		
		≥ N 1 stage	43 (45.7)		
	M 1 stage		19 (20.2)		0 (0.0)
	Grading	1	1 (1.1)		0 (0.0)
		2	56 (59.6)		
		3	37 (39.4)		
	Resection margin	0	61 (64.9)		0 (0.0)
		1	26 (27.7)		
		2	7 (7.4)		
		≥ R1	33 (35.1)		
	AJCC/UICC classification (8^th^ ed.)	Ia	6 (6.4)		2 (2.1)
		Ib	15 (16.0)		
		IIa	16 (17.0)		
		IIb	24 (25.5)		
		III	12 (12.8)		
		IV	19 (20.2)		
		≥ III	31 (33.0)		

*ASA* American Society of Anesthesiologists, *CT* computed tomography, *MRI* magnetic resonance imaging, *PRBC* packed red blood cells, *FFP* fresh frozen plasma, *AJCC* American Joint Committee on Cancer, *UICC* Union for International Cancer Control

### Pancreatic surgery

Distal pancreatectomy was performed in 85 (90.4%) patients. Subtotal pancreatectomy was performed in 9 (9.6%) patients and was neither associated with increased postoperative morbidity (i.e., complications with or without the need of surgical revision; *p* ≥ 0.050) nor inferior survival (14.36 versus 12.42 months; *p* = 0.869).

Simultaneous splenectomy was performed in all but one patient, who underwent splenectomy after polytrauma prior to the pancreatic resection.

**Fig. 1:**
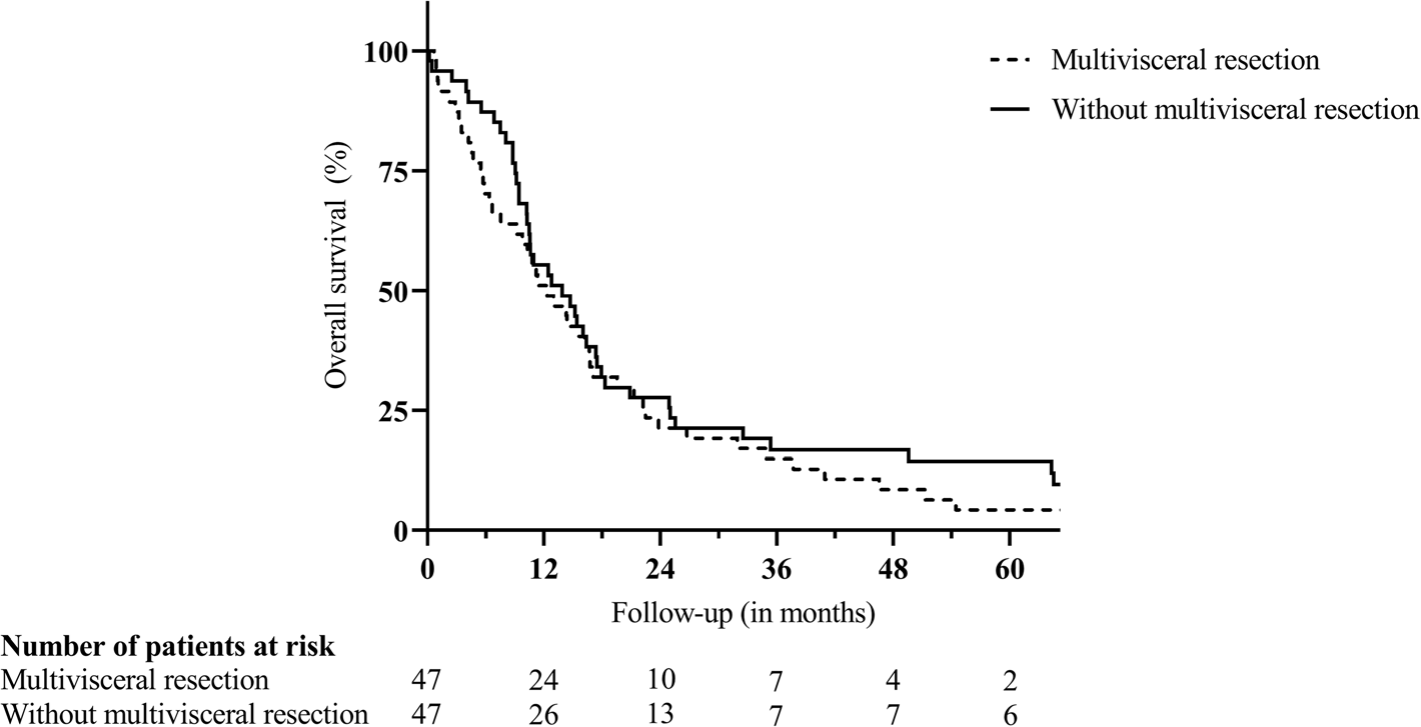
Postoperative survival after standard distal pancreatectomy or multivisceral resection for ductal adenocarcinoma. Legend: *p* = 0.377

**Fig. 2:**
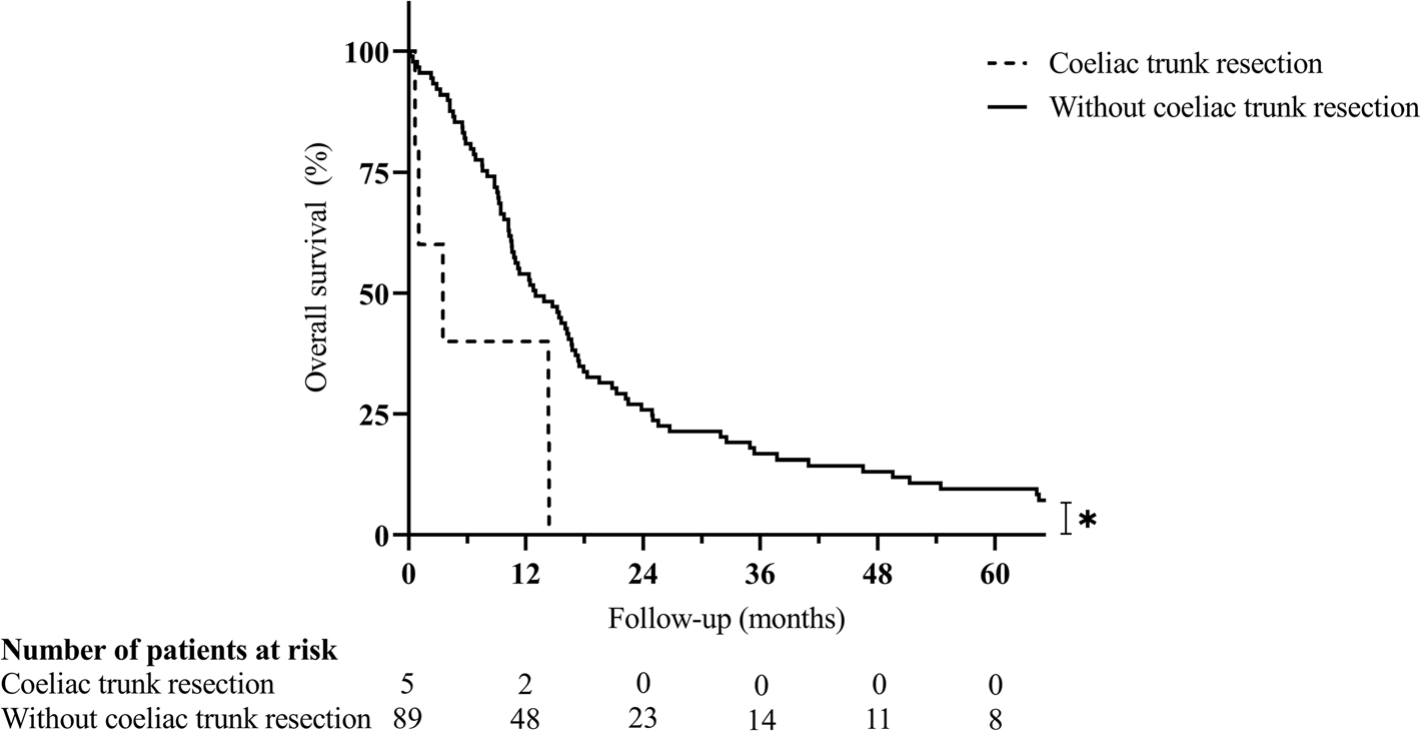
Survival after distal pancreatectomy for ductal adenocarcinoma in case of coeliac trunk resection. Legend: *p = 0.012. Statistical significance (*p* < 0.050) is indicated with an asterisk

**Fig. 3:**
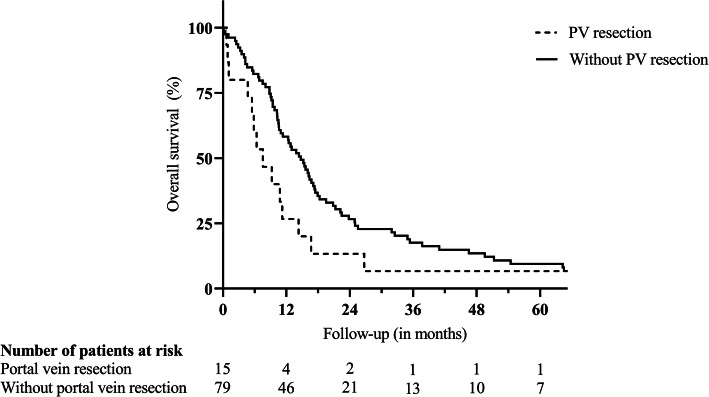
Survival after distal pancreatectomy for ductal adenocarcinoma in case of portal vein resection. Legend: *p* = 0.064. *PV* portal vein

Due to the invasion of extrapancreatic tissue, 47 (50.0%) patients underwent multivisceral resections, including partial resections of the liver (12 patients), the large and small intestine (12 and 8 patients, respectively), the stomach (14 patients), the left kidney (5 patients), the left adrenal gland (13 patients), the portal vein (15 patients), and the coeliac trunk (5 patients). Of note, in 21 (44.7%) of these patients, preoperative imaging led to the suspected diagnosis of local invasion of neighboring tissue or distant metastases prior surgery, whereas in 24 (51.1%) patients, extrapancreatic disease was diagnosed intraoperatively (missing information in 2 (4.3%) patients). Supplementary Table 1 (Additional file 1) gives an overview of the performed resections. Patients undergoing multivisceral resection were of similar mean age (64.02 versus 66.04 years; *p* = 0.296), but displayed a significantly lower rate of ASA scores > 2 (16.2 versus 39.0%; *p* = 0.023) when compared to patients with standard resection. Mean operation time (188.37 versus 142.87 min; *p* < 0.001), rate of intraoperative blood transfusions (53.2 versus 19.1%; *p* = 0.001), and mean number of intraoperatively transfused packed red blood cell (PRBC) units (2.23 versus 0.45 units; *p* < 0.001) were significantly elevated in case of multivisceral resection. Postoperative complications requiring surgical revision were observed more frequently after multivisceral resections (14.9 versus 2.1%; *p* = 0.029), whereas the incidence of other postoperative complications was comparable to all other patients (31.9 versus 38.3%; *p* = 0.517). Clinically relevant pancreatic fistulas were slightly more frequent after multivisceral resections (30.4 versus 17.4%; *p* = 0.143) and significantly more frequent after simultaneous partial colectomy (50.0 versus 20.0%; *p* = 0.034). Accordingly, multivisceral resections led to a significantly prolonged median postoperative hospital stay (18 versus 13 days; *p* = 0.007). Despite an increase in postoperative morbidity, the postoperative survival after multivisceral resection was not inferior (12.35 versus 13.87 months; *p* = 0.377; Fig. 1) with two patients still alive after 5 years. However, (partial) resections of the coeliac trunk (3.52 versus 13.01 months; *p* = 0.012; Fig. 2) or the portal vein (7.56 versus 14.72 months; *p* = 0.064; Fig. 3) were associated with a trend towards inferior survival. (Table 2).

**Table 2:** Postoperative outcome of the study cohort after distal pancreatectomy

Variables		n_**abs**_ (n_**%**_)	Mean; Median (Range)	Missing values n (%)
Hospital stay (in days)			19.33; 15 (5-109)	0 (0.0)
Complications requiring surgery		8 (8.5)		0 (0.0)
Complications not requiring surgery		33 (35.1)		
Pancreatic fistula	Not measured	47 (50.0)		2 (2.1)
	No biochemical leak	17 (18.1)		
	Biochemical leak (former Grade A)	6 (6.4)		
	Grade B	17 (18.1)		
	Grade C	5 (5.3)		
	≥ Grade B	22 (23.4)		
Postoperative days until removal of drains			12.75; 9 (3-46)	3 (3.2)
Pancreatin post-surgery		34 (36.2)		7 (7.4)
In-hospital mortality		6 (6.4)		0 (0.0)
Follow-up time in months			23.18; 12.90 (0.16-220.92)	0 (0.0)
Survival in months (Kaplan-Meier)			27.96; 12.78 (n.a.)	0 (0.0.)
1-year survival (Kaplan-Meier)		n.a. (53.2)		
3-year survival (Kaplan-Meier)		n.a. (15.8)		
5-year survival (Kaplan-Meier)		n.a. (9.0)		
Deceased at time of analysis		88 (93.6)		0 (0.0)

**Fig. 4:**
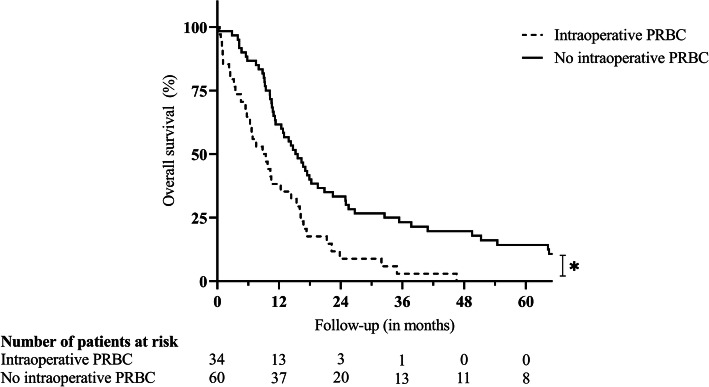
Survival after distal pancreatectomy for ductal adenocarcinoma in case of intraoperative transfusion of PRBC. Legend: *p < 0.001. Statistical significance (*p* < 0.050) is indicated with an asterisk. *PRBC* packed red blood cells

Intraoperative blood transfusions were necessary in 34 (36.2%) patients, ranging from 1 to 24 units of PRBC, and were significantly associated with inferior survival (8.81 versus 15.21 months; *p* < 0.001; Fig. 4). Of note, preoperative anemia was detected in 14 (14.9%) patients. Neither the rate of intraoperative blood transfusions (57.1 versus 33.3%; *p* = 0.083) nor the mean number of intraoperatively transfused PRBC units (1.50 versus 1.35 units; *p* = 0.072) were significantly elevated in these patients.

Of note, none of the surgical procedures was carried out minimally invasive.

Table 1 provides further information on surgical details.

### Histopathological results

Locally advanced tumor stage (T stage ≥ 3) was observed in 50 (53.2%) patients and resulted in inferior survival (9.76 versus 17.35 months; *p* = 0.039).

**Fig. 5:**
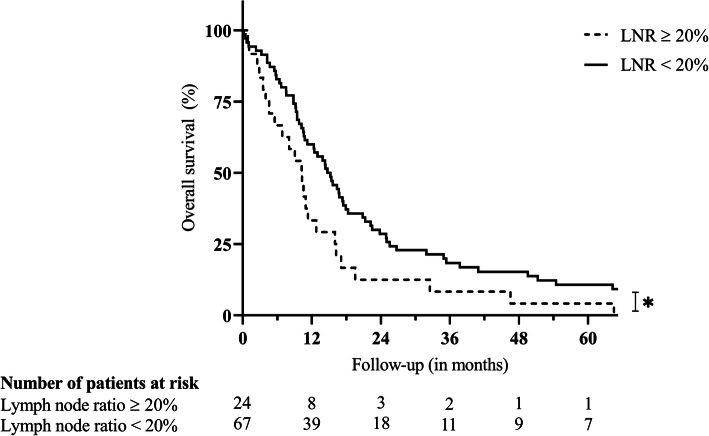
Survival after distal pancreatectomy for adenocarcinoma in case of lymph node ratio ≥ 20%. Legend: *p = 0.027. Statistical significance (*p* < 0.050) is indicated with an asterisk. *LNR* lymph node ratio

Lymph node metastases were identified in 43 (45.7%) cases. Positive nodal status (N1/N2) was significantly associated with inferior survival (10.55 versus 15.64 months; *p* = 0.015) as were positive lymph node ratios of ≥ 20% (10.25 versus 14.72 months; *p* = 0.027; Fig. 5).

Distant metastases were diagnosed in 19 (20.2%) patients and did not significantly impact on postoperative survival (10.78 versus 13.87 months; *p* = 0.217), whereas advanced AJCC/UICC stages (≥ 3; 31 (33.0%) patients) were significantly associated with inferior survival (10.55 versus 15.21; *p* = 0.010).

Positive resection margins (R1/R2) were confirmed in 33 (35.1%) patients and were also significantly associated with inferior survival (10.22 versus 15.64 months; *p* = 0.004).

Table 1 summarizes the histopathological data of the study cohort.

### Postoperative outcome

Median follow-up after pancreatic resection was 12.90 (0.16–220.92) months.

Patients stayed in hospital for a median of 15 (5–109) days.

We observed postoperative complications in 36 (38.3%) patients. Surgical revision was required in 8 (8.5%) cases. Clinically relevant pancreatic fistulas were observed in 22 (23.4%) patients resulting in a prolonged removal of abdominal drains (17 patients) or surgical revision (5 patients).

Six (6.4%) patients died in the postoperative course. Three patients died due to severe bleeding (two patients) and gastric perforation with consecutive sepsis (one patient) as a result of pancreatic fistula. One patient died after severe bleeding caused by a postoperative intraabdominal abscess, and two further patients died after pulmonary embolism with consecutive multiple organ failure.

The median estimated survival was 12.78 months with 1-, 3-, and 5-year survival rates of 53.2%, 15.8%, and 9.0%. Eighty-eight (93.6%) patients were deceased at the time of analysis. Table 2 summarizes selected variables regarding the postoperative course and outcome of the study cohort.

### Risk factors for clinically relevant pancreatic fistulas

Univariable regression analysis identified simultaneous partial colectomy as risk factor for the incidence of clinically relevant pancreatic fistulas (OR = 4.000; CI 95% = 1.138–14.063; *p* = 0.031); however, none of the analyzed variables was an independent risk factor in multivariable analysis (Supplementary Table 2, Additional file 1).

### Risk factors for postoperative survival

The results of the univariable Cox regression analysis are displayed in Table 3. Multivariable analysis identified coeliac trunk resection (HR = 3.364; CI 95% = 1.147–9.861; *p* = 0.027), portal vein resection (HR = 2.275; CI 95% = 1.221–4.236; *p* = 0.010), intraoperative transfusion of PRBC (HR = 1.998; CI 95% = 1.229–3.247; *p* = 0.005), and lymph node ratio in percentage (HR = 1.022; CI 95% = 1.009–1.034; *p* = 0.001) as independent risk factors for postoperative survival.

**Table 3:** Cox regression analysis for identification of risk factors for survival after distal pancreatectomy

Variables			Univariable analysis			Multivariable analysis		
			HR	CI 95%	*p* value	HR	CI 95%	*p* value
Biometrics	Female gender		0.770	0.506–1.171	0.222			
	Age (in years)		1.003	0.980–1.027	0.805			
Preoperative course	Initial symptoms	Epigastric pain	0.946	0.592–1.512	0.817			
		Weight loss	1.202	0.777–1.861	0.408			
		Back pain	1.489	0.890–2.491	0.130			
		Inappetence	1.512	0.817–2.798	0.188			
		Nausea	0.702	0.362–1.362	0.296			
		Vomiting	0.881	0.356–2.182	0.785			
		Fatigue	**3.007**	**1.279**–**7.071**	**0.012**			
		Others	1.060	0.666–1.687	0.806			
	ASA score > 2		1.362	0.823–2.255	0.229			
	Diabetes		1.236	0.740–2.066	0.419			
	Diagnostics	Hemoglobin (in g/dl)	0.973	0.821–1.154	0.756			
		Anemia	1.442	0.793–2.621	0.231			
		Platelets (in 10^3^ per μl)	**0.996**	**0.993**–**1.000**	**0.047**			
		Elevated CA19-9	1.480	0.853–2.568	0.163			
		Elevated CEA	0.848	0.478–1.504	0.572			
Surgical details	Type of pancreatectomy	More than distal	1.060	0.530–2.121	0.869			
	Multivisceral resection		1.208	0.794–1.839	0.378			
	Including (partial) resection of	Liver	1.386	0.750–2.562	0.298			
		Large intestine	1.207	0.654–2.228	0.548			
		Small intestine	1.198	0.577–2.485	0.628			
		Stomach	1.184	0.665–2.107	0.567			
		Kidney	1.581	0.636–3.928	0.324			
		Adrenal gland	1.120	0.608–2.060	0.717			
		Coeliac trunk	**3.091**	**1.226**–**7.797**	**0.017**	**3.364**	**1.147–9.861**	**0.027**
		Portal vein	1.711	0.962–3.044	0.067	**2.275**	**1.221–4.236**	**0.010**
	Intraoperative PRBC		**2.235**	**1.429**–**3.498**	**< 0.001**	**1.998**	**1.229–3.247**	**0.005**
	Intraoperative PRBC (*n*)		**1.160**	**1.080**–**1.245**	**< 0.001**			
	Intraoperative FFP		**1.808**	**1.024**–**3.191**	**0.041**			
	Operation time (in minutes)		**1.004**	**1.000**–**1.009**	**0.033**			
Histopathological results	Tumor localization	Including body	1.053	0.691–1.604	0.811			
	Invasion of peripancreatic tissue		**2.148**	**1.182**–**3.904**	**0.012**			
	T staging	Tumor size (in cm)	1.060	0.983–1.143	0.132			
		T stage ≥ 3	**1.558**	**1.019**–**2.381**	**0.040**			
	Lymph node status	Lymph nodes (*n*, total)	0.985	0.958–1.012	0.271			
		Lymph nodes (*n*, positive)	**1.201**	**1.065**–**1.355**	**0.003**			
		Lymph node ratio (in %)	**1.020**	**1.008**–**1.033**	**0.002**	**1.022**	**1.009–1.034**	**0.001**
		≥ N 1 stage	**1.688**	**1.100**–**2.592**	**0.017**			
		N 2 stage	**2.095**	**1.027**–**4.274**	**0.042**			
	M 1 stage		1.381	0.825–2.311	0.219			
	Grading	Grading > 2	1.266	0.826–1.939	0.279			
	Resection margin	≥ R1	**1.892**	**1.219**–**2.935**	**0.004**			
		R 2 status	1.008	0.464–2.189	0.984			
	AJCC/UICC classification (8th ed.)	≥ III	**1.815**	**1.149**–**2.868**	**0.011**			

Bold values indicate statistical significance (*p* < 0.050)

*ASA* American Society of Anesthesiologists, *PRBC* packed red blood cells, *FFP* fresh frozen plasma, *AJCC* American Joint Committee on Cancer, *UICC* Union for International Cancer Control

## Discussion

Patients suffering from pancreatic adenocarcinoma continue to have an extremely poor prognosis. In cases of unresectable disease, median survival of around 7 months has been reported, and although surgical resection is regarded as the only chance for long-term survival, 5-year survival rates after resection of up to around 20% are still unsatisfying [1, 20–22]. Studies investigating prognostic factors for postoperative survival often include patients irrespective of the exact tumor localization, although abundant data suggests that lesions of the pancreatic head differ not only in terms of local tumor extent, invasion of adjacent tissue, and necessary surgical strategy to achieve negative resection margins, but also in tumor biology [21–26].

In order to optimize preoperative patient selection, we have evaluated the effects of multivisceral resections, among other variables, on the postoperative outcome in patients undergoing distal or subtotal pancreatectomy for ductal adenocarcinoma. In summary, multivisceral resections in general were associated with increased morbidity and the risk of reoperation, but not with increased short- or long-term mortality. Although there are currently no meta-analyses on the matter, a large multi-center study by Paye et al. and previously published single-center reports support our observations [10, 11, 27–30]. Of note, most of these publications included resections for different types of pancreatic tumors, whereas the current study focused explicitly on histologically confirmed pancreatic ductal adenocarcinoma.

The rate of arterial and venous resections in our patient cohort was comparable to previous publications; however, reports on the effects of vascular resections in case of distal pancreatectomy are conflicting [10, 27]. In general, the need for arterial resections to achieve tumor-free resection margins in case of pancreatic adenocarcinoma is seen critical or even as a contraindication for surgery, as it is associated with increased morbidity and mortality [13]. In the current study, resection of the coeliac trunk was associated with a considerable increase in postoperative mortality with two of five patients dying in the postoperative course and a resulting median survival of only 3.52 months. Nonetheless, a recent systematic review and meta-analysis by Gong et al. revealed that distal pancreatectomy with en bloc celiac axis resection can result in favorable survival, despite increased postoperative morbidity, and improved quality of life in selected patients, especially when compared to palliative treatment [31].

As a result of refined surgical techniques, simultaneous resections of the portal and/or the superior mesenteric vein are generally regarded as safe and can facilitate long-term survival [13, 32]. Data on the effect of such venous resections in case of distal pancreatectomy is scarce, and recommendations result mostly from reports on pancreatic head carcinoma [33–36]. Interestingly, recent meta-analyses as well as large single center studies have shown rather discouraging results [35–37]. In the current study, simultaneous resection of the portal vein resulted in a median survival of only 7.56 months and only one patient survived more than 5 years. Previous publications did not identify resections of the mesenteric-portal axis as risk factors for survival in case of distal pancreatectomy; however, missing details of the extent of venous resection or mainly minor resections (such as wedge or tangential resections) limit the significance and comparability of such observations [11, 27]. In our study, all but one patient underwent segmental resections of the portal vein due to macroscopically suspected invasion. Such resections, unlike tangential resections, were recently identified as an independent risk factor for postoperative survival after resection of pancreatic adenocarcinoma by Serenari et al. [38].

Further prognostic factors identified in the current study were intraoperative blood transfusions, which were demonstrated to result in adverse oncologic effects such as shorter disease-free survival in the past, and a higher lymph node ratio [39]. The latter has been confirmed by several authors including a systematic review for pancreatic adenocarcinoma in general. Thus, apart from the classical nodal status, the lymph node ratio should be an inherent part when planning and optimizing postoperative adjuvant strategies [23, 40–45].

The incidence of clinically relevant pancreatic fistulas was comparable to reports of the past [46]. Although we observed a trend towards more fistulas in patients after multivisceral resection (30.4%) and especially after simultaneous partial colectomy (50.0%), multivariable analysis did not identify extended surgery as an independent risk factor. This is consistent with reports from previous single-center studies [30, 47]. Recently, Ecker et al. analyzed 2026 consecutive distal pancreatectomies and identified risk factors such as young age, obesity, non-malignant histology, or concomitant splenectomy, among others; however, the authors concluded that despite the considerable size of the study, postoperative pancreatic fistulas cannot be reliably predicted, since individual surgeon performance, applied techniques, and patient-specific pancreatic texture limit the significance of clinical applicability of mentioned observations [46].

Despite long follow-up durations—only one surviving patient was followed up less than 5 years—the retrospective nature of the study and the limited number of patients hamper definitive conclusions. Further limitations of our study are missing information on disease recurrence and systemic therapy, especially as the latter is of increasing importance in a more and more multimodal approach for pancreatic cancer [48, 49]. Although reliable data on this matter is still scarce, a review of resections in patients with oligometastatic pancreatic disease underlines the importance of standardizing neoadjuvant and adjuvant chemo(radio)therapy strategies and defines a response towards neoadjuvant systemic treatment as important patient selection criterion along with an adequate performance status as well as resectability of the primary tumor and the metastases [50]. Larger patient cohorts, meta-analyses, and prospective trials are urgently required to confirm our observations in this lethal disease.

## Conclusions

Our data indicates that multivisceral resection in case of advanced left-sided pancreatic adenocarcinoma is justified, as it is not associated with increased mortality and can even facilitate long-term survival, albeit with an increase in postoperative morbidity. However, vascular resections are associated with a dismal prognosis and should be performed after thorough consideration and only in selected patients.

## Supplementary information

**Additional file 1: Supplementary Table 1.** Overview of the different types of resections in the investigated cohort. **Supplementary Table 2.** Logistic regression analysis identifying risk factors for clinically relevant pancreatic fistulas after distal pancreatectomy.

**Additional file 2: Supplementary Table 3.1.** Overview of patients undergoing standard distal pancreatectomy including selected variables sorted by year of surgery. **Supplementary Table 3.2.** Overview of patients undergoing multivisceral resection including selected variables sorted by year of surgery.

## Data Availability

The datasets used and/or analyzed during the current study are available from the corresponding author on reasonable request.

## Abbreviations

AJCC: American Joint Committee on Cancer
ASA: American Society of Anesthesiologists
UICC: Union for International Cancer Control
CA19-9: Carbohydrate antigen 19-9
CEA: Carcinoembryonic antigen
PRBC: Packed red blood cells
OR: Odds ratio
HR: Hazard ratio
CI: Confidence interval
PV: Portal vein
LNR: Lymph node ratio
