# Supplementary material for: Multivisceral resection for adenocarcinoma of the pancreatic body and tail—a retrospective single-center analysis
Source: World J Surg Oncol. 2020 Aug 20;18:218. doi: 10.1186/s12957-020-01973-x (PMC7441692; doi:10.1186/s12957-020-01973-x)
Supplement: Supplementary file 1 — Additional file 1: Supplementary Table 1. Overview of the different types of resections in the investigated cohort. Supplementary Table 2. Logistic regression analysis identifying risk factors for clinically relevant pancreatic fistulas after distal pancreatectomy. [file 12957_2020_1973_MOESM1_ESM.docx]

**Supplementary Table 1.** Overview of the different types of resections in the investigated cohort.

| **Types of resection** | | **n** |
| --- | --- | --- |
| Left pancreatic resection + splenectomy **without** multivisceral resection | | **47** |
| Left pancreatic resection + splenectomy **with** multivisceral resection | | **46** |
|  | + gastrectomy | 2 |
|  | + gastrectomy + resection of coeliac trunk + omentum majus resection | 1 |
|  | + gastrectomy + resection of coeliac trunk + colectomy + left adrenalectomy + left-sided nephrectomy + omentum majus resection | 1 |
|  | + gastrectomy + colectomy | 1 |
|  | + gastrectomy + colectomy + left adrenalectomy + left-sided nephrectomy | 1 |
|  | + gastrectomy + colectomy + small bowel resection + liver resection | 1 |
|  | + gastrectomy + left adrenalectomy + left-sided nephrectomy + liver resection | 1 |
|  | + gastrectomy + portal vein resection + small bowel resection + resection of mesocolon | 1 |
|  | + gastrectomy + liver resection + small bowel resection | 1 |
|  | + gastrectomy + liver resection + resection of mesocolon | 1 |
|  | + gastrectomy + liver resection | 1 |
|  | + gastrectomy + omentum majus resection | 1 |
|  | + colectomy + left adrenalectomy | 2 |
|  | + colectomy + left adrenalectomy + left-sided nephrectomy | 2 |
|  | + colectomy + left adrenalectomy + left sided nephrectomy + resection of coeliac trunk + portal vein resection | 1 |
|  | + colectomy + left adrenalectomy + liver resection | 1 |
|  | + colectomy + omentum majus resection | 1 |
|  | + colectomy + small bowel resection | 1 |
|  | + portal vein resection | 10 |
|  | + portal vein resection + liver resection | 2 |
|  | + portal vein resection + resection of coeliac trunk | 1 |
|  | + small bowel resection | 2 |
|  | + small bowel resection + left adrenalectomy | 1 |
|  | + small bowel resection + resection of mesocolon | 1 |
|  | + liver resection | 3 |
|  | + liver resection + resection of mesocolon | 1 |
|  | + resection of coeliac trunk | 1 |
|  | + left adrenalectomy | 2 |
|  | + resection of omentum majus | 1 |
| Left pancreatic resection - splenectomy **with** multivisceral resection | | **1** |
|  | + gastrectomy + left adrenalectomy | 1 |

**Supplementary Table 2.** Logistic regression analysis identifying risk factors for clinically relevant pancreatic fistulas after distal pancreatectomy.

| **Variables** | | | | **Univariable Analysis** | | | **Multivariable Analysis** | | |
| --- | --- | --- | --- | --- | --- | --- | --- | --- | --- |
|  |  |  |  | **OR** | **CI-95%** | **p-value** | **OR** | **CI-95%** | **p-value** |
| **Biometrics** | Female gender | | | 0.787 | 0.301-2.058 | 0.625 |  |  |  |
|  | Age (in years) | | | 1.005 | 0.955-1.058 | 0.854 |  |  |  |
| **Preoperative Course** | Initial symptoms | | Epigastric pain | 0.866 | 0.305-2.459 | 0.787 |  |  |  |
|  |  |  | Weight loss | 1.968 | 0.742-5.220 | 0.174 |  |  |  |
|  |  |  | Back pain | 0.313 | 0.066-1.485 | 0.144 |  |  |  |
|  |  |  | Inappetence | 1.407 | 0.387-5.121 | 0.604 |  |  |  |
|  |  |  | Nausea | 1.611 | 0.434-5.980 | 0.476 |  |  |  |
|  |  |  | Vomiting | 3.316 | 0.618-17.800 | 0.162 |  |  |  |
|  |  |  | Fatigue | 1.550 | 0.264-9.106 | 0.628 |  |  |  |
|  |  |  | Others | 1.592 | 0.586-4.324 | 0.361 |  |  |  |
|  | ASA score > 2 | | | 1.025 | 0.338-3.107 | 0.965 |  |  |  |
|  | Diabetes | | | 1.420 | 0.469-4.298 | 0.535 |  |  |  |
|  | Diagnostics | | Hemoglobin (in g/dl) | 1.095 | 0.761-1.577 | 0.625 |  |  |  |
|  |  |  | Anemia | 1.333 | 0.373-4.765 | 0.658 |  |  |  |
|  |  |  | Platelets (in 10³ per µl) | 0.996 | 0.990-1.003 | 0.268 |  |  |  |
|  |  |  | Elevated CA19-9 | 0.656 | 0.196-2.198 | 0.495 |  |  |  |
|  |  |  | Elevated CEA | **11.250** | **2.803-45.156** | **0.001** |  |  |  |
| **Surgical details** | Type of pancreatectomy | | More than distal | n.a. | n.a. | n.a. |  |  |  |
|  | Multivisceral resection | | | 2.078 | 0.774-5.580 | 0.147 |  |  |  |
|  | Including (partial) resection of | Liver | | 0.255 | 0.031-2.100 | 0.204 |  |  |  |
|  |  | Large intestine | | **4.000** | **1.138-14.063** | **0.031** |  |  |  |
|  |  | Small intestine | | 1.067 | 0.199-5.707 | 0.940 |  |  |  |
|  |  | Stomach | | 1.506 | 0.415-5.471 | 0.534 |  |  |  |
|  |  | Kidney | | 5.368 | 0.836-34.488 | 0.077 |  |  |  |
|  |  | Adrenal gland | | 2.279 | 0.660-7.873 | 0.193 |  |  |  |
|  |  | Coeliac trunk | | 2.233 | 0.349-14.312 | 0.397 |  |  |  |
|  |  | Portal vein | | 1.765 | 0.531-5.865 | 0.354 |  |  |  |
|  | Intraoperative PRBC | | | 1.597 | 0.603-4.228 | 0.346 |  |  |  |
|  | Intraoperative PRBC (n) | | | 1.239 | 0.987-1.555 | 0.065 |  |  |  |
|  | Intraoperative FFP | | | 1.765 | 0.531-5.865 | 0.354 |  |  |  |
|  | Operation time (in minutes) | | | 1.006 | 0.998-1.015 | 0.138 |  |  |  |
| **Histopathological results** | Tumor localization | | Including body | 1.216 | 0.460-3.213 | 0.693 |  |  |  |
|  | Invasion of peripancreatic tissue | | | 0.931 | 0.267-3.247 | 0.911 |  |  |  |
|  | T staging | Tumor size (in cm) | | 1.062 | 0.868-1.299 | 0.559 |  |  |  |
|  |  | T stage ≥ 3 | | 1.750 | 0.652-4.695 | 0.266 |  |  |  |
|  | Lymph node status | Lymph nodes (n total) | | 0.988 | 0.929-1.051 | 0.696 |  |  |  |
|  |  | Lymph nodes (n positive) | | 1.058 | 0.831-1.346 | 0.648 |  |  |  |
|  |  | Lymph node ratio (in %) | | 1.015 | 0.990-1.040 | 0.240 |  |  |  |
|  |  | ≥ N 1 stage | | 1.432 | 0.545-3.761 | 0.466 |  |  |  |
|  |  | N2 stage | | 1.632 | 0.372-7.154 | 0.516 |  |  |  |
|  | M 1 stage | | | 0.338 | 0.071-1.601 | 0.172 |  |  |  |
|  | Grading | Grading > 2 | | 0.857 | 0.318-2.310 | 0.761 |  |  |  |
|  | Resection margin | ≥ R1 | | 0.790 | 0.285-2.190 | 0.650 |  |  |  |
|  |  | R 2 status | | 0.508 | 0.058-4.465 | 0.541 |  |  |  |
|  | AJCC/UICC classification (8^th^ ed.) | ≥III | | 0.506 | 0.166-1.539 | 0.230 |  |  |  |

Bold values indicate statistical significance (p < 0.050). *ASA* American Society of Anesthesiologists, *PRBC* packed red blood cells, *FFP* fresh frozen plasma, *AJCC* American Joint Committee on Cancer, *UICC* Union for International Cancer Control
