# Supplementary material for: Multivisceral resection for adenocarcinoma of the pancreatic body and tail—a retrospective single-center analysis
Source: World J Surg Oncol. 2020 Aug 20;18:218. doi: 10.1186/s12957-020-01973-x (PMC7441692; doi:10.1186/s12957-020-01973-x)
Supplement: Supplementary file 2 — Additional file 2: Supplementary Table 3.1. Overview of patients undergoing standard distal pancreatectomy including selected variables sorted by year of surgery. Supplementary Table 3.2. Overview of patients undergoing multivisceral resection including selected variables sorted by year of surgery. [file 12957_2020_1973_MOESM2_ESM.docx]

**Supplementary Table 3.1.** Overview of patients undergoing standard distal pancreatectomy including selected variables sorted by year of surgery.

| **Year of surgery** | **Patient Number** | **Multivisceral resection** | **Coeliac trunk resection** | **Portal vein resection** | **Intraoperative PRBC** | **Intraoperative PRBC (n)** | **Lymph node ratio (%)** | **Operation time (min)** | **Surgeon** |
| --- | --- | --- | --- | --- | --- | --- | --- | --- | --- |
| 1995 | 1 | 0 | 0 | 0 | 0 | 0 | 0 |  |  |
| 1999 | 2 | 0 | 0 | 0 | 1 | 3 | 13 | 265 | B |
| 2000 | 3 | 0 | 0 | 0 | 1 | 2 | 0 | 170 | B |
|  | 4 | 0 | 0 | 0 | 1 | 1 | 7 | 205 | B |
| 2001 | 5 | 0 | 0 | 0 | 0 | 0 | 0 | 125 | A |
| 2003 | 6 | 0 | 0 | 0 | 1 | 2 | 31 | 270 | A |
|  | 7 | 0 | 0 | 0 | 0 | 0 | 0 | 125 | A |
| 2004 | 8 | 0 | 0 | 0 | 0 | 0 | 0 | 120 | A |
|  | 9 | 0 | 0 | 0 | 0 | 0 | 33 | 135 | B |
| 2005 | 10 | 0 | 0 | 0 | 0 | 0 |  | 68 | A |
|  | 11 | 0 | 0 | 0 | 0 | 0 | 17 | 220 | B |
|  | 12 | 0 | 0 | 0 | 0 | 0 | 0 | 185 | B |
|  | 13 | 0 | 0 | 0 | 0 | 0 | 33 | 96 | A |
| 2006 | 14 | 0 | 0 | 0 | 0 | 0 | 20 | 155 | A |
|  | 15 | 0 | 0 | 0 | 0 | 0 | 0 | 104 | A |
|  | 16 | 0 | 0 | 0 | 0 | 0 | 18 | 150 | B |
|  | 17 | 0 | 0 | 0 | 1 | 3 | 0 | 160 | B |
|  | 18 | 0 | 0 | 0 | 1 | 4 | 42 | 179 | B |
| 2007 | 19 | 0 | 0 | 0 | 0 | 0 | 29 | 93 | A |
|  | 20 | 0 | 0 | 0 | 0 | 0 | 53 | 150 | B |
|  | 21 | 0 | 0 | 0 | 0 | 0 | 33 | 150 | A |
|  | 22 | 0 | 0 | 0 | 0 | 0 | 0 | 101 | A |
| 2008 | 23 | 0 | 0 | 0 | 0 | 0 | 86 | 107 | A |
|  | 24 | 0 | 0 | 0 | 1 | 2 | 0 | 160 | A |
|  | 25 | 0 | 0 | 0 | 0 | 0 | 0 | 224 | B |
| 2009 | 26 | 0 | 0 | 0 | 1 | 2 | 0 | 117 | A |
|  | 27 | 0 | 0 | 0 | 0 | 0 | 25 | 195 | B |
| 2010 | 28 | 0 | 0 | 0 | 0 | 0 | 23 | 101 | A |
|  | 29 | 0 | 0 | 0 | 0 | 0 |  | 211 | A |
|  | 30 | 0 | 0 | 0 | 0 | 0 | 0 | 101 | A |
|  | 31 | 0 | 0 | 0 | 0 | 0 | 0 | 89 | A |
|  | 32 | 0 | 0 | 0 | 0 | 0 | 10 | 107 | A |
| 2012 | 33 | 0 | 0 | 0 | 1 | 2 | 23 | 201 | A |
|  | 34 | 0 | 0 | 0 | 0 | 0 | 0 | 136 | B |
|  | 35 | 0 | 0 | 0 | 0 | 0 | 0 | 105 | B |
| 2013 | 36 | 0 | 0 | 0 | 0 | 0 | 7 | 104 | B |
|  | 37 | 0 | 0 | 0 | 0 | 0 | 13 | 121 | B |
|  | 38 | 0 | 0 | 0 | 0 | 0 | 0 | 112 | A |
|  | 39 | 0 | 0 | 0 | 0 | 0 | 0 | 110 | A |
| 2014 | 40 | 0 | 0 | 0 | 0 | 0 | 0 | 152 | B |
|  | 41 | 0 | 0 | 0 | 0 | 0 | 19 | 174 | B |
| 2015 | 42 | 0 | 0 | 0 | 0 | 0 | 17 | 117 | A |
| 2016 | 43 | 0 | 0 | 0 | 0 | 0 | 25 | 111 | A |
|  | 44 | 0 | 0 | 0 | 0 | 0 | 14 | 96 | A |
|  | 45 | 0 | 0 | 0 | 0 | 0 | 5 | 161 | B |
|  | 46 | 0 | 0 | 0 | 0 | 0 | 0 | 105 | A |
|  | 47 | 0 | 0 | 0 | 0 | 0 | 0 | 129 | A |

*PRBC* packed red blood cells, Surgeon *A* main surgeon, Surgeon *B* other surgeons

**Supplementary Table 3.2.** Overview of patients undergoing multivisceral resection including selected variables sorted by year of surgery.

| **Year of surgery** | **Patient Number** | **Multivisceral resection** | **Coeliac trunk resection** | **Portal vein resection** | **Intraoperative PRBC** | **Intraoperative PRBC (n)** | **Lymph node ratio (%)** | **Operation time (min)** | **Surgeon** |
| --- | --- | --- | --- | --- | --- | --- | --- | --- | --- |
| 1995 | 48 | 1 | 0 | 0 | 0 | 0 | 0 |  |  |
| 1996 | 49 | 1 | 0 | 0 | 0 | 0 | 33 | 220 | B |
| 1997 | 50 | 1 | 0 | 0 | 1 | 2 | 13 | 210 | B |
|  | 51 | 1 | 0 | 0 | 1 | 2 | 0 | 170 | B |
| 1998 | 52 | 1 | 0 | 0 | 1 | 3 | 33 | 285 | B |
| 1999 | 53 | 1 | 0 | 1 | 1 | 3 | 100 | 215 | A |
| 2000 | 54 | 1 | 1 | 1 | 1 | 10 | 6 | 360 | B |
| 2001 | 55 | 1 | 0 | 0 | 0 | 0 | 0 | 125 | B |
|  | 56 | 1 | 0 | 0 | 1 | 3 | 0 | 280 | A |
|  | 57 | 1 | 1 | 0 | 1 | 3 | 33 | 115 | A |
| 2004 | 58 | 1 | 0 | 0 | 0 | 0 | 20 | 130 | B |
|  | 59 | 1 | 0 | 0 | 1 | 3 | 0 | 228 | A |
| 2005 | 60 | 1 | 0 | 0 | 1 | 4 | 0 | 192 | B |
|  | 61 | 1 | 1 | 1 | 0 | 0 |  | 185 | A |
| 2006 | 62 | 1 | 0 | 1 | 1 | 2 | 25 | 155 | A |
|  | 63 | 1 | 0 | 1 | 0 | 0 | 0 | 210 | A |
|  | 64 | 1 | 0 | 1 | 1 | 3 | 0 | 180 | A |
|  | 65 | 1 | 0 | 1 | 0 | 0 | 0 | 185 | B |
|  | 66 | 1 | 0 | 0 | 0 | 0 | 0 | 150 | B |
| 2007 | 67 | 1 | 0 | 1 | 0 | 0 | 0 | 149 | A |
| 2008 | 68 | 1 | 0 | 0 | 0 | 0 | 0 | 165 | A |
|  | 69 | 1 | 0 | 1 | 0 | 0 | 10 | 165 | B |
|  | 70 | 1 | 0 | 0 | 0 | 0 | 33 | 145 | A |
|  | 71 | 1 | 0 | 0 | 1 | 4 | 0 | 204 | B |
|  | 72 | 1 | 0 | 1 | 1 | 2 | 0 | 110 | B |
| 2009 | 73 | 1 | 0 | 0 | 1 | 6 | 44 | 190 | A |
|  | 74 | 1 | 1 | 0 | 1 | 5 | 0 | 263 | B |
| 2010 | 75 | 1 | 0 | 0 | 1 | 3 | 25 | 154 | A |
|  | 76 | 1 | 0 | 0 | 1 | 2 | 0 | 130 | B |
|  | 77 | 1 | 0 | 1 | 0 | 0 | 0 | 176 | B |
| 2011 | 78 | 1 | 0 | 0 | 0 | 0 | 0 | 150 | B |
|  | 79 | 1 | 0 | 0 | 1 | 3 | 30 | 268 | B |
|  | 80 | 1 | 0 | 0 | 0 | 0 | 21 | 155 | B |
| 2012 | 81 | 1 | 0 | 0 | 1 | 2 | 0 | 204 | A |
|  | 82 | 1 | 0 | 0 | 0 | 0 | 0 | 159 | A |
|  | 83 | 1 | 0 | 0 | 1 | 2 | 0 | 151 | A |
|  | 84 | 1 | 0 | 1 | 0 | 0 | 14 | 173 | A |
|  | 85 | 1 | 0 | 1 | 0 | 0 | 0 | 145 | B |
| 2013 | 86 | 1 | 0 | 0 | 0 | 0 | 0 | 242 | B |
|  | 87 | 1 | 0 | 0 | 1 | 3 | 0 | 232 | B |
| 2014 | 88 | 1 | 0 | 1 | 0 | 0 | 6 | 176 | B |
|  | 89 | 1 | 0 | 0 | 0 | 0 | 0 | 138 | B |
| 2015 | 90 | 1 | 0 | 0 | 0 | 0 | 4 | 220 | B |
|  | 91 | 1 | 0 | 0 | 1 | 1 | 0 | 174 | A |
|  | 92 | 1 | 0 | 1 | 1 | 3 | 0 | 154 | A |
| 2016 | 93 | 1 | 0 | 0 | 1 | 7 | 0 | 177 | A |
|  | 94 | 1 | 1 | 0 | 1 | 24 | 17 | 301 | B |

*PRBC* packed red blood cells, Surgeon *A* main surgeon, Surgeon *B* other surgeons
